# Effects of recombinant LH supplementation to recombinant FSH during induced ovarian stimulation in the GnRH-agonist protocol: a matched case-control study

**DOI:** 10.1186/1477-7827-7-58

**Published:** 2009-06-04

**Authors:** José G Franco, Ricardo LR Baruffi, João Batista A Oliveira, Ana L Mauri, Claudia G Petersen, Paula Contart, Valeria Felipe

**Affiliations:** 1 Center for Human Reproduction Prof Franco Junior, Ribeirão Preto, SP, Brazil; 2 Department of Gynecology and Obstetrics, Botucatu Medical School, São Paulo State University, UNESP, Brazil

## Abstract

**Background:**

Some studies have suggested that the suppression of endogenous LH secretion does not seem to affect the majority of patients who are undergoing assisted reproduction and stimulation with recombinant FSH (r-FSH). Other studies have indicated that a group of normogonadotrophic women down-regulated and stimulated with pure FSH preparations may experience low LH concentrations that compromise the IVF parameters. The present study aimed to compare the efficacy of recombinant LH (r-LH) supplementation for controlled ovarian stimulation in r-FSH and GnRH-agonist (GnRH-a) protocol in ICSI cycles.

**Methods:**

A total of 244 patients without ovulatory dysfunction, aged <40 years and at the first ICSI cycle were divided into two groups matched by age according to an ovarian stimulation scheme: Group I (n = 122): Down-regulation with GnRH-a + r-FSH and Group II (n = 122): Down-regulation with GnRH-a + r-FSH and r-LH (beginning simultaneously).

**Result(s):**

The number of oocytes collected, the number of oocytes in metaphase II and fertilization rate were significantly lower in the Group I than in Group II (*P *= 0.036, *P *= 0.0014 and *P *= 0.017, respectively). In addition, the mean number of embryos produced per cycle and the mean number of frozen embryos per cycle were statistically lower (*P *= 0.0092 and *P *= 0.0008, respectively) in Group I than in Group II. Finally the cumulative implantation rate (fresh+thaw ed embryos) was significantly lower (*P *= 0.04) in Group I than in Group II. The other clinical and laboratory results analyzed did not show difference between groups.

**Conclusion:**

These data support r-LH supplementation in ovarian stimulation protocols with r-FSH and GnRH-a for assisted reproduction treatment.

## Background

The pharmacology of ovarian stimulation has been strongly influenced by the two-cell, two-gonadotrophin theory [1] while, historically, follicular stimulation protocols have included both luteinizing hormone (LH) and follicle-stimulating hormone (FSH) in an attempt to mimic normal physiology [2]. During recent years, the effect of LH on follicular maturation and pregnancy outcome during the course of ovarian stimulation in relation to assisted reproduction has received increasing attention. This interest reflects the fact that modern stimulation protocols have resulted in substantially lower LH concentrations than those observed in the natural cycle and in previously used protocols. The introduction of gonadotrophin-releasing hormone agonists (GnRH-a) in the mid-1980s successfully circumvented the problems of a premature LH surge. There has also been a gradual shift from human gonadotrophin (HMG) with equal amounts of FSH and LH-like activity over pure urine-derived FSH preparations to recombinant human FSH (r-FSH), without LH activity [3].

Some studies have suggested that the suppression of endogenous LH secretion does not seem to affect the majority of patients who are undergoing assisted reproduction and stimulation with r-FSH. Other studies have indicated that a group of normogonadotrophic women down-regulated and stimulated with pure FSH preparations may experience low LH concentrations that compromise the parameters of the IVF treatment [3].

According to current concepts in folliculogenesis, LH plays an essential role in the final stages of follicular maturation [4,5]. Once an appropriate stage of follicular development has been achieved in response to FSH treatment, granulosa cells become receptive to LH stimulation, and LH becomes capable of exerting its actions on both theca cells and granulosa cells. In fact, at non-saturating concentrations of FSH and LH, the response is additive. Moreover, it has been postulated that the maturing follicle reduces its dependence on FSH by acquiring LH receptors [4,5]. Thus, LH may play an essential role in determining oocyte maturity and development potential in IVF cycles. However, exposure of the developing follicle to inappropriately high concentrations of LH may interfere with follicular and oocyte maturation and thus adversely affect the reproductive process [6,7].

The present study aims to compare the efficacy of recombinant LH (r-LH) supplementation in women undergoing assisted reproduction and stimulation with r-FSH in the GnRH-a protocol of ovarian stimulation in ICSI cycles.

## Methods

This was a retrospective, observational study including a total of 244 patients without any ovulatory dysfunction, aged <40 years and at the first ICSI cycle. The data were collected from the medical records at the Centre for Human Reproduction Prof Franco Jr. between January 2005 and July 2007. All the patients signed terms of informed consent before the fertilization treatment granting use of their data for scientific purposes, with the procedure being approved by the local Research Ethics Committee. No other procedure, except those required for treating infertility, was carried out. The decisions to include or exclude r-LH in the ovarian stimulation followed the protocol of the Clinic: until July 2006, no cycles included r-LH; after this date, all cycles included r-LH.

Routinely, first pituitary down-regulation was established with nafarelin acetate at the dose of 400 μg/day (Synarel^®^; Pharmacia, SP, Brazil.), started during the second phase of the previous cycle. After 14 days of treatment with GnRH-a, r-FSH (Gonal F^®^; Serono, SP, Brazil) was started. Then the patients were divided into two groups matched by age:

-Group I (n = 122): r-FSH was administered at the fixed dose of 150–225 IU for a period of 7 days. On the 8th day of ovarian stimulation, follicular development started to be monitored by vaginal ultrasound only, and r-FSH doses were adapted according to ovarian response. No r-LH was used.

-Group II (n = 122): r-LH (Luveris^®^, Serono, SP, Brazil) was administered at 75 IU/day with an r-FSH fixed dose (150–225 IU) for a period of 7 days. On the 8th day of ovarian stimulation, the r-FSH dose was adapted according to ovarian response, and r-LH supplementation was increased to 150 IU/day when one or more follicles measuring ≥ 10 mm in diameter were found.

Then, when one or more follicles measuring ≥ 17 mm were observed, r-hCG (Ovidrel^®^, Serono, SP, Brazil) was administered at the dose of 250 μg.

Oocytes were retrieved from the follicles by ultrasound guided transvaginal puncture 36–38 h after r-hCG administration. The oocytes retrieved were stripped from the cumulus-corona cells utilizing a solution containing 40 IU/ml of hyaluronidase (Irvine Scientific, USA) and the denuded oocytes were classified by maturity based on morphological criteria. Oocytes were incubated in P1 with 10% HAS (Irvine Scientific, USA) until the moment for ICSI. Discontinuous gradients of Isolate^® ^(Irvine Scientific, USA) were used to separate spermatozoa from the seminal fluid in the 40–90% fractions. ICSI were performed as described previously [8,9].

Embryos were routinely transferred after 48 h in culture with a Frydman catheter (Frydman^® ^Classic Catheter 4.5 CCD Laboratoire C.C.D; Paris, France) guided by abdominal ultrasound using a 3.5 MHz convex transducer (Aloka SSD-1100; Aloka Co. Ltd, Tokyo, Japan). Supernumerary embryos were cryopreserved at the end of the 2^nd ^day. The same physician performed all transfers. All patients received luteal phase supplementation with vaginal natural progesterone (Utrogestan^®^; Farmoquímica, RJ, Brazil).

For the freezing-thawing process, an embryo freeze-thaw media kit (Irvine Scientific, USA) was used. Frozen thawed embryo transfer was performed after assessment of embryo cleavage, when the division of at least one of the blastomeres was observed after 24 hours of culturing. Only one protocol was used for transferring frozen-thawed embryos. Estradiol valerate (Cicloprimogyna^®^; Schering, SP, Brazil) was administered from the first day to day 14 of the cycle at a daily dose of 6 mg. Progesterone (Utrogestan^®^; Farmoquímica, RJ, Brazil) was also introduced vaginally on day 14 at 400 mg/day, as long as endometrial thickness was ≥ 6 mm [10] and was increased to a daily dose of 800 mg on the day of embryo transfer. Thawing was performed on day 5 of progesterone treatment and embryos were transferred on day 6. The same physician performed all transfers.

Data were analyzed using InStat version 3.0 (GraphPad Software, San Diego, California, USA) on a Macintosh computer (Apple Computer Inc., Cupertino, California, USA). The Student's t, Mann-Whitney and x^2 ^tests were utilized when appropriate. The level of significance was set at *P *< 0.05.

## Results

The general characteristics of the study population are summarized in Table 1. There were 122 subjects assigned to each of the two treatment groups. The age of patients in Group I (32.7 ± 3.7 y) was equal (*P *= 0.99) to that of patients in Group II (32.7 ± 3.7 y). An equal distribution (*P *> 0.05) of the other main characteristics was also observed for groups I and II.

**Table 1:** General characteristics of the study population

	Total	Group I (no r-LH)	Group II (r-LH)	P
Female age	32.7 ± 3.7	32.7 ± 3.7	32.7 ± 3.7	ns
Distribution of females				
<30 y	19.7 (48)	19.7 (24)	19.7 (24)	ns
30–35 y	54.1(132)	54.1 (66)	54.1 (66)	
36–39 y	26.2 (61)	26.2 (32)	26.2 (32)	
Male age	37.2 ± 6.8	37.2 ± 6.3	37.1 ± 7.2	ns
Infertility Factor				
-Male factor	45.5(111)	48.4 (59)	42.6 (52)	ns
-Idiopathic	16.8 (41)	14.0 (17)	19.7 (24)	
-Endometriosis	13.5 (33)	9.8 (12)	17.2 (21)	
-Tubal–peritoneal	13.5 (33)	17.2 (21)	9.8 (12)	
-Tubal–peritoneal+endometriosis	4.9 (12)	5.7 (7)	4.1 (5)	
-Tubal–peritoneal + male	2.9 (7)	3.3 (4)	2.5 (3)	
-Endometriosis + male	2.9 (7)	1.6 (2)	4.1 (5)	
BMI (kg/m^2^)	23.2 ± 2.9	23.1 ± 3.0	23.4 ± 2.9	ns
Infertility				
-Primary	77 (188)	77.9 (95)	76.2 (93)	ns
-Secondary	23 (56)	22.1 (27)	23.8 (29)	
Duration of infertility (y)	4.6 ± 3.5	5.2 ± 4.1	4.0 ± 3.0	ns
basal FSH (mUI/ml)	5.9 ± 2.6	5.2 ± 1.9	6.4 ± 3.1	ns
basal LH (mUI/ml)	5.4 ± 3.4	4.8 ± 2.6	6.1 ± 4.1	ns
basal Estradiol (pg/ml)	47.7 ± 23.5	48.3 ± 28.8	47.1 ± 23.6	ns

ns: not significant

Values are mean ± SD or %(n)

The total dose of r-FSH was similar (*P *= 0.13) between Group I (2181 ± 719 IU) and Group II (2032 ± 651 IU). The number of oocytes collected from Group I (9.3 ± 4.7) was statistically lower (*P *= 0.036) than that in Group II (10.9 ± 5.9). In addition, the number of oocytes in metaphase II was significantly lower (*P *= 0.0014) in Group I (6.7 ± 4.1) in relation to Group II (8.5 ± 4.6), but the number of immature oocytes was not significantly different between the two groups (Group I:2.6 ± 2.7; Group II:2.4 ± 2.8, *P *= 0.12). Also, the fertilization rate was significantly lower (*P *= 0.017) in Group I (66.2%, 634/958) than in Group II (71.1%, 835/1175). The mean number of embryos produced per cycle was statistically lower (*P *= 0.0092) in Group I (5.4 ± 3.7) than in Group II (6.5 ± 3.0). The mean number of embryos transferred from Group I (2.0 ± 0.8) was similar (P = 0.42) to that from Group II (2.0 ± 0.5). The mean number of embryos frozen per cycle was statistically lower (*P *= 0.0008) in Group I (3.0 ± 3.6) than in Group II (4.4 ± 3.8). The implantation rate was similar (*P *= 0.17) between Group I (17.9%) and Group II (23.2%). The pregnancy rate per patient, cycle and transfer were not statistically significant different for Group I (29.5%, 29.5%, and 31.6%, respectively) compared with those of Group II (33.6%, 33.6%, and 34.4%, respectively). The miscarriage rate was similar (*P *= 0.90) between Group I (11.1%) and Group II (14.6%). The cumulative live birth rate per cycle was statically similar (*P *= 0.65) between the two groups (Group I: 23.8%; Group II: 27%). Table 2 displays these data.

**Table 2:** Clinical and laboratory results of the fresh ICSI procedures.

	Group I (No r-LH)	Group II (r-LH)	p-value
Patients (n)	122	122	
Cycles (n)	122	122	
Transfers (n)	114	119	
Total dose r-FSH (IU)	2181 ± 719	2032 ± 651	ns
Total dose r-LH (IU)	------	1048 ± 262	
Retrieved oocytes (n)	9.3 ± 4.7	10.9 ± 5.9	0.036
Immature oocytes (n)	2.6 ± 2.7	2.4 ± 2.8	ns
Metaphase II oocytes (n)	6.7 ± 4.1	8.5 ± 4.6	0.0014
Fertilization rate	66.2%	71.1%	0.017
Embryos/cycle(n)	5.4 ± 3.7	6.5 ± 3.0	0.0092
Embryos transferred(n)	2.0 ± 0.8	2.0 ± 0.5	ns
Frozen embryos/cycle(n)	3.0 ± 3.6	4.4 ± 3.8	0.0008
Implantation rate	17.9%	23.2%	ns
Pregnancy rate			
-per patient	29.5%	33.6%	ns
-per transfer	31.6%	34.4%	ns
Miscarriage rate	11.1%	14.6%	ns
Live birth/patient (or started cycle)	23.8%	27.0%	ns

ns: not significant

Table 3 shows laboratory and clinical data with respect to the freezing-thawing process. The cumulative implantation rate (fresh + thawed embryos) was significantly lower (*P *= 0.04) in Group I (14.7%) than in Group II (20.6%). At the moment, the cumulative pregnancy rate per transfer and patient (fresh + thawed) are similar (*P *= 0.31 and *P *= 0.24 respectively) for Group I (27.9% and 37.7%, respectively) and Group II (33.5% and 45.6%, respectively). Also, the cumulative live birth rate per cycle and patient were statically similar (*P *= 0.54 and *P *= 0.57, respectively) between the two groups (per cycle:Group I: 20.2%, Group II: 23.5%; per patient: Group I: 28.7%, Group II: 32.8%)

**Table 3:** Clinical and laboratory characteristics of the frozen/thawed ICSI procedures

	Group I (no r-H)	Group II (r-LH)	p-value
Patients (frozen + thawed) (n)	43	40	
Transfers (n)	51	48	
Thawed and transferred embryos (n)	128	114	
Implantation rate/thawed embryos	8.6%	15%	ns
Pregnancy rate			
-per thawed embryo transfer	19.6%	31.2%	ns
-per patient	23.2%	37.5%	ns
Cumulative implantation rate (fresh + thawed)	14.7%	20.6%	0.04
Cumulative pregnancy rate (fresh+ thawed)			
-per transfer	27.9%	33.5%	ns
-per patient	37.7%	45.9%	ns
Cumulative live births/(fresh+ thawed)			
-per started cycle	20.2%	23.5%	ns
-per patient	28.7%	32.8%	ns

ns: not significant

When age subgroup analyses comparing results from women aged ≤ 35 y with those >35 y were carried out (Table 4), it was observed that the difference found between the groups with and without r-LH was due primarily to the results in the subgroups of patients aged ≤ 35 y. The number of oocytes in metaphase II was significantly lower (*P *= 0.003) in the subgroup without r-LH aged ≤ 35 y (7.4 ± 4.3) than in the subgroup with r-LH aged ≤ 35 y (9.3 ± 4.5). Also, the fertilization rate was significantly lower (*P *= 0.04) in the subgroup without r-LH aged ≤ 35 y (67.8%, 533/786) than in the one with r-LH aged ≤ 35 y (72.5%, 687/948). In addition, the mean number of embryos produced per cycle was statistically lower (*P *= 0.003) in the subgroup ≤ 35 y without r-LH (5.9 ± 3.7) than the one of the same age range with r-LH (7.6 ± 4.1). The only exception was the mean number of embryos frozen per cycle which presented a difference both between the subgroups aged ≤ 35 y (subgroup without r-LH:3.7 ± 3.9; subgroup with r-LH:5.0 ± 3.8, *P *= 0.008) and between the subgroups aged >35 y (subgroup without r-LH:1.1 ± 2.1; subgroup with r-LH:2.8 ± 3.3, P = 0.03). The other evaluations did not show any difference among the subgroups. Table 5 shows laboratory and clinical data with respect to the freezing-thawing process in relation to subgroups. Again, the only difference found was in the cumulative implantation rate (fresh + thawed embryos), which was significantly lower (*P *= 0.008) in the subgroup without r-LH aged ≤ 35 y (14.2%) when compared to the subgroup including the same age range with r-LH (23.2%). The other evaluations found no differences among the subgroups.

**Table 4:** Female age subgroup analysis ≤ 35 years vs. >35 years.

	Group I (No r-LH)		Group II (r-LH)	
	≤ 35 years	> 35 years	≤ 35 years	> 35 years
Patients	90	32	90	32
Cycles	90	32	90	32
Transfers	84	30	89	30
Total dose r-FSH (IU)	1976.7 ± 583.3	2756.3 ± 760.6	1873.6 ± 546.2	2477.3 ± 721.8
Total dose r-LH (IU)	------	------	1034.2 ± 245.9	1087.5 ± 302.4
Retrieved oocytes	10.4 ± 4.7	6.4 ± 3.4	11.9 ± 5.8	8.1 ± 5.2
Immature oocytes	3.0 ± 2.9	1.6 ± 1.4	2.6 ± 2.9	1.9 ± 2.5
Metaphase II Oocyte	7.4 ± 4.3^a^	4.8 ± 3.0	9.3 ± 4.5^a^	6.7 ± 4.5
Fertilization rate	67.8%^b^	58.7%	72.5%^b^	65.2%
Embryos/cycle (n)	5.9 ± 3.7^c^	3.2 ± 2.0	7.6 ± 4.1^c^	4.6 ± 3.2
embryos transferred (n)	2.0 ± 0.7	2.1 ± 0.9	2.0 ± 0.3	2.0 ± 0.9
frozen embryos per cycle(n)	3.7 ± 3.9^d^	1.1 ± 2.1^e^	5.0 ± 3.8^d^	2.8 ± 3.3^e^
Implantation rate	18.3%	16.7%	25.8%	15.4%
Pregnancy rate				
-per patient	27.8%	34.4%	36.7%	25%
-per transfer	29.8%	36.7%	37.1%	26.7%
Miscarriage rate	8%	18.2%	15.2%	12.5%
Live birth/patient (or started cycle	24.4%	21.9%	28.9%	21.9%

Clinical and laboratory results of the fresh ICSI procedures.

^a-b-c-d-e ^Values within rows with the same superscript letter were significantly different: *P *< 0.05

**Table 5:** Female age subgroup analysis ≤ 35 years vs. > 35 years.

	Group I (no r-H)		Group II (r-LH)	
	≤ 35 years	> 35 years	≤ 35 years	> 35 years
Patients (frozen + thawed)	36	7	30	10
Transfers (n)	44	7	37	11
Thawed and transferred embryos (n)	115	13	85	29
Implantation rate per thawed embryos	7.8%	15.4%	17.6%	6.9%
Pregnancy rate				
-per thawed embryo transfer	18.2%	28.6%	35.1%	18.2%
-per patient	22.2%	28.6%	43.3%	10%
Cumulative implantation rate (fresh + thawed)	14.2%^a^	16.4%	23.2%^a^	14.9%
Cumulative pregnancy rate (fresh+thawed)				
-per transfer	25.8%	35.1%	36.5%	24.4%
-per patient	36.7%	40.6%	51.1%	31.2%
Cumulative live birth (fresh+thawed)				
-per started cycle	20.1%	20.5%	25.2%	18.6%
-per patient	30%	25%	35.5%	25%

Clinical and laboratory characteristics of the frozen/thawed ICSI procedures.

^a ^*P *< 0.05

## Discussion

The validity of the two-cell, two-gonadotrophin hypothesis, which suggests that both LH and FSH are required for ovarian steroidogenesis in a gonadotrophin-deficient population (World Health Organization classification I), is clear. However, there is still considerable controversy on the need for additional LH supplementation in cycles of assisted reproduction techniques using GnRH-a [11].

It has been widely demonstrated that, during ovarian stimulation with FSH and concomitant administration of a GnRH-a, the endogenous LH level decreases, reaching its lowest value during the late stimulation phase. Thus, it would seem logical that if LH supplementation is to show any benefit, it would occur at least in the late follicular phase.

Oliveira et al. [12] compared, by meta-analysis, the efficacy of r-LH supplementation for controlled ovarian stimulation in r-FSH and GnRH-a protocol. The data showed fewer days of stimulation, less total amount of r-FSH administered and higher serum estradiol levels on the day of hCG administration with the r-LH supplementation protocol. However, differences were not observed in the number of oocytes retrieved, number of mature oocytes, and clinical pregnancy per oocyte retrieved, implantation or miscarriage rates. Moreover, in this meta-analysis, all randomized controlled trials started r-LH after 6 days of r-FSH.

On the other hand, our data showed more significant positive effects of r-LH: a larger number of retrieved oocytes and oocytes in metaphase II, elevated fertilization rate and increased numbers of embryos frozen and produced by cycle. Nevertheless, r-LH supplementation (75UI) was started with r-FSH protocol at the beginning of the follicular phase. Normally, LH contributes to the ovarian stimulation process when granulosa cells of the follicles become receptive to LH, and follicles ≥ 10 mm with LH receptors keep growing due to decreasing FSH concentrations in the mid to late follicular phase [13]. Willis et al. [14] showed that granulosa cells from ovulatory patients (with either normal ovaries or polycystic ovaries) responded to LH once follicles reached 9.5/10 mm. In contrast, granulosa cells from anovulatory women with polycystic ovaries responded to LH in follicles smaller than 4 mm. Thus, the precocity of r-LH administration could be beneficial for follicles that are ≥ 10 mm during the first days of r-FSH use (day 1 to day 7). For this reason, we opted for early LH initiation in the present study (first day of stimulation).

However, the LH requirement for the maintenance of follicular steroidogenesis is likely to be low because <1% of follicular receptors need to be occupied to permit normal steroidogenesis [15]. The number of patients with profound LH depression (after GnRH-a) differs among the published studies [16]. Severe LH deficiency was reported as 6% by Loumaye et al. [17], 40% by Fleming et al. [18] and 49% by Westegaard et al. [19], with each research group using different criteria and assays for definition. Westergaad et al. [19] determined that very low LH concentrations (< 0.5 IU/l) can also be detrimental to fertility outcomes, showing that this group of patients suffers more often from early pregnancy loss when compared with patients having normal LH concentrations (> 0.5 IU/l) (45% versus 9%, respectively). Ruvolo et al. [20] showed data suggesting that supplementation with r-LH during the follicular phase improves some laboratory parameters, and, in particular, seems to significantly reduce the number of immature oocytes collected after pick-up. The apoptosis rate in cumulus cells was higher in the control group than in the r-LH group. This finding may represent a situation in which, despite the presence of a low quantity of this hormone because of the paracrine effect mediated by secreting factors in the theca and oocyte cells, the cumulus cells are preserved from apoptosis. By maintaining their physiological function for a longer time, cumulus cells are better able to support nuclear and cytoplasmic maturation of the oocyte until ovulation. Bosco et al. [21] demonstrated that apoptosis in human oocytes determines fertilization failure after ICSI. If the activation of apoptosis in the oocyte is regulated by molecular signals coming from cumulus cells through gap junctions, the lower apoptotic rate in cumulus cells would be an indicator of good oocyte quality in relation to a greater capacity to be fertilized and to produce embryos of higher potential.

Therefore, there is a need for further clinical research to establish appropriate clinical criteria for LH supplementation. FSH plays a crucial role in recruitment, selection, and dominance, while LH contributes to dominance, maturation, and ovulation. However, Dumerin et al. [22] showed that pretreatment with r-LH followed by r-FSH in women who were profoundly down-regulated by GnRH agonist (Depot) increased small antral follicles and normally fertilized (2PN) embryos. This suggests possible beneficial effects of up-regulated androgen upon follicular metabolism and an early action. On the other hand, there is basic, experimental and clinical evidence unequivocally indicating that ovarian follicles have development-related requirements for stimulation by LH. There is a "threshold" for LH requirements during folliculogenesis [23]. Whereas each follicle has a threshold beyond which it must be stimulated by FSH to initiate preovulatory development, it may also have a "ceiling" below which it should be stimulated by LH. Therefore, LH concentrations should be neither too high nor too low during stimulation of ovulation in order to not compromise reproductive performance.

On the other hand, multiple novel roles of LH have been proposed and it has been postulated that LH may affect IVF/ICSI results both by determining oocyte quality and by influencing uterine receptivity via ovarian estradiol secretion or through direct effects on the endometrium, myometrium, and uterine artery and vein [24-26]. In addition, our results from fresh embryo transfer with respect to implantation rates and clinical pregnancies are superior, but not significantly greater in the r-LH supplementation group. However, the cumulative implantation rate (fresh + thawed embryos) is significantly higher in the patients with r-LH supplementation.

An age subgroup analysis in order to detect a beneficial effect of LH supplementation in a particular age range was carried out, as had been done in some studies showing a positive result of this practice in patients >35 y[27,28]. Nevertheless, our analysis showed a diverse result, evidencing greater benefit from the use of r-LH in patients aged ≤ 35 y. However, the sample size is too small, particularly in the patient subgroup >35 y (total 64 patients), to draw conclusions on this point with sufficient statistical power.

We acknowledge the limitations of the current study, firstly the difference in the time at which the two groups underwent their respective procedures. It is impossible to control for increased experience and potential improvements in the clinical and laboratory settings that could influence the results in such a study. However, besides the laboratory procedures having been exactly the same in the two groups, the responsible laboratory staff had already accumulated vast experience, having worked with IVF/ICSI for more than 10 years before the commencement of the study. Second, although the patients were matched by age, the make-up of patients by diagnoses in each group was quite diverse and this in itself could have affected some of the differences observed between groups I and II. However, it should be emphasized that the statistical analysis did not show any difference between the two groups. Finally, to evaluate the efficacy of the two protocols considering both fresh and thawed ET, more clinical endpoints such as live birth rate per started cycle present greater importance than the started cycle implantation rate. However, it should be noted that avoiding a beta-type error when assessing a difference between the two study groups (maintaining the same rates of live birth rate herein described), with a power of 80% and a level of significance of 5%, would require a sample size of more the 2500 cycles in each group.

In conclusion, the data of the present study support r-LH supplementation in ovarian stimulation protocols with r-FSH and GnRH-a for assisted reproduction treatment.

## Competing interests

The authors declare that they have no competing interests.

## Authors' contributions

JGF was responsible for designing and coordinating the study. All authors were responsible for data collection, data analysis, and data interpretation of the manuscript. RLRB, JBA and JGF were responsible for the statistical work and for writing of the manuscript. JGF was responsible for reviewing the manuscript. All authors read and approved the final manuscript.
